# A Case of Pituitary Abscess and Multiple Cerebral Intracranial Aneurysms Related to Dental Caries

**DOI:** 10.7759/cureus.87708

**Published:** 2025-07-11

**Authors:** Genki Ikuta, Yasuyuki Kaku, Naoki Shinojima, Takamasa Mizuno, Akitake Mukasa

**Affiliations:** 1 Department of Neurosurgery, Kumamoto University Hospital, Kumamoto, JPN; 2 Department of Neurosurgery, Ariake Medical Center, Kumamoto, JPN

**Keywords:** anterior communicating artery aneurysm, anterior wall aneurysm of the internal carotid artery, endovascular treatment, intracranial infectious aneurysm, pituitary abscess, true posterior communicating artery aneurysm, dental caries

## Abstract

We report a rare case of a 60-year-old woman who initially presented with subarachnoid hemorrhage and was later diagnosed with a pituitary abscess 11 months after coil embolization. Poor oral hygiene and cultures positive for *Escherichia coli* (*E. coli*) from both the throat and pituitary abscess pus suggested that the infection had a dental origin. Because of the rarity of aneurysms with atypical morphology and location, the previously treated multiple intracranial aneurysms-including an anterior communicating artery aneurysm, a true posterior communicating artery aneurysm, and an anterior wall aneurysm of the internal carotid artery-were retrospectively suspected to be infectious. Because infectious aneurysms of the anterior communicating artery are exceptionally uncommon, we reviewed similar reported cases. This case highlights the potential for dental infections to cause serious central nervous system complications. Clinicians should maintain a high index of suspicion for pituitary abscess and the possibility of infectious aneurysms when encountering multiple aneurysms with atypical morphology or location, particularly in patients with poor oral hygiene or a suspected Rathke’s cleft cyst.

## Introduction

Pituitary abscesses and infectious intracranial aneurysms are rare infectious conditions [1,2]. Pituitary abscesses account for approximately 1% of all pituitary lesions and are typically associated with infections such as meningitis, sinusitis, osteomyelitis of the sella turcica, cavernous sinus thrombosis, and sepsis. Additionally, they may arise from preexisting lesions such as Rathke’s cleft cysts [1]. Infectious intracranial aneurysms represent 0.5% to 6.5% of all intracranial aneurysms and are commonly caused by systemic infections, including infective endocarditis, meningitis, and dental infections [2]. Dental caries are widespread and generally considered benign; however, in rare instances, they can be a source of serious infectious intracranial complications. We report a rare case of a pituitary abscess presumed to be caused by an infection that originated from dental caries and was accompanied by multiple intracranial aneurysms that were retrospectively suspected to be infectious.

## Case presentation

**Figure 1:**
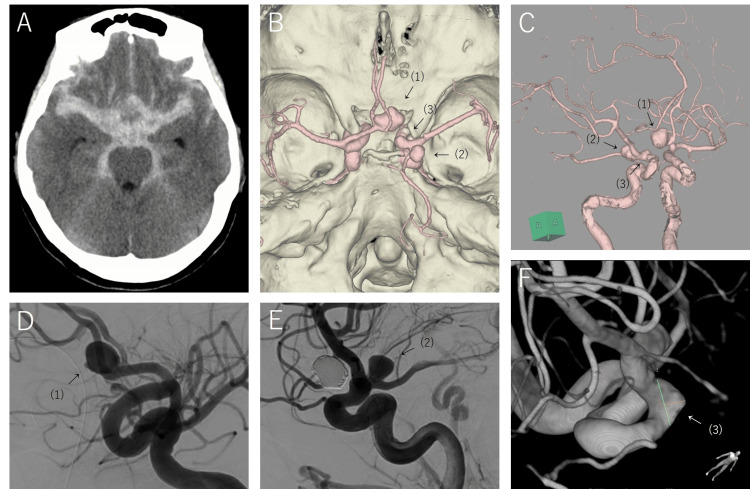
(A) Initial brain computed tomography revealed subarachnoid hemorrhage. (B, C) Computed tomography angiography showed an anterior communicating artery aneurysm (1), a true posterior communicating artery aneurysm (2), and an anterior wall aneurysm of the internal carotid artery (3). (D) Preoperative angiography of the anterior communicating artery aneurysm. (E) Preoperative angiography of the true posterior communicating artery aneurysm. (F) Preoperative angiography of the anterior wall aneurysm of the internal carotid artery.

A 60-year-old woman presented to the emergency department of our hospital with a headache and disturbance of consciousness. She had a history of hypertension and many untreated dental caries. She had no history of smoking and no known allergies and reported occasional alcohol consumption. She was able to independently perform activities of daily living and lived with her husband. Her only contact with animals involved her cat. The patient was disoriented during examination. Computed tomography revealed a subarachnoid hemorrhage. Computed tomography angiography revealed multiple intracranial aneurysms. A ruptured anterior communicating artery aneurysm was treated using coil embolization. The remaining aneurysms, including the true posterior communicating artery aneurysm and an anterior wall aneurysm of the internal carotid artery, were treated at postoperative month (POM) 1 (Figure 1).

**Figure 2:**
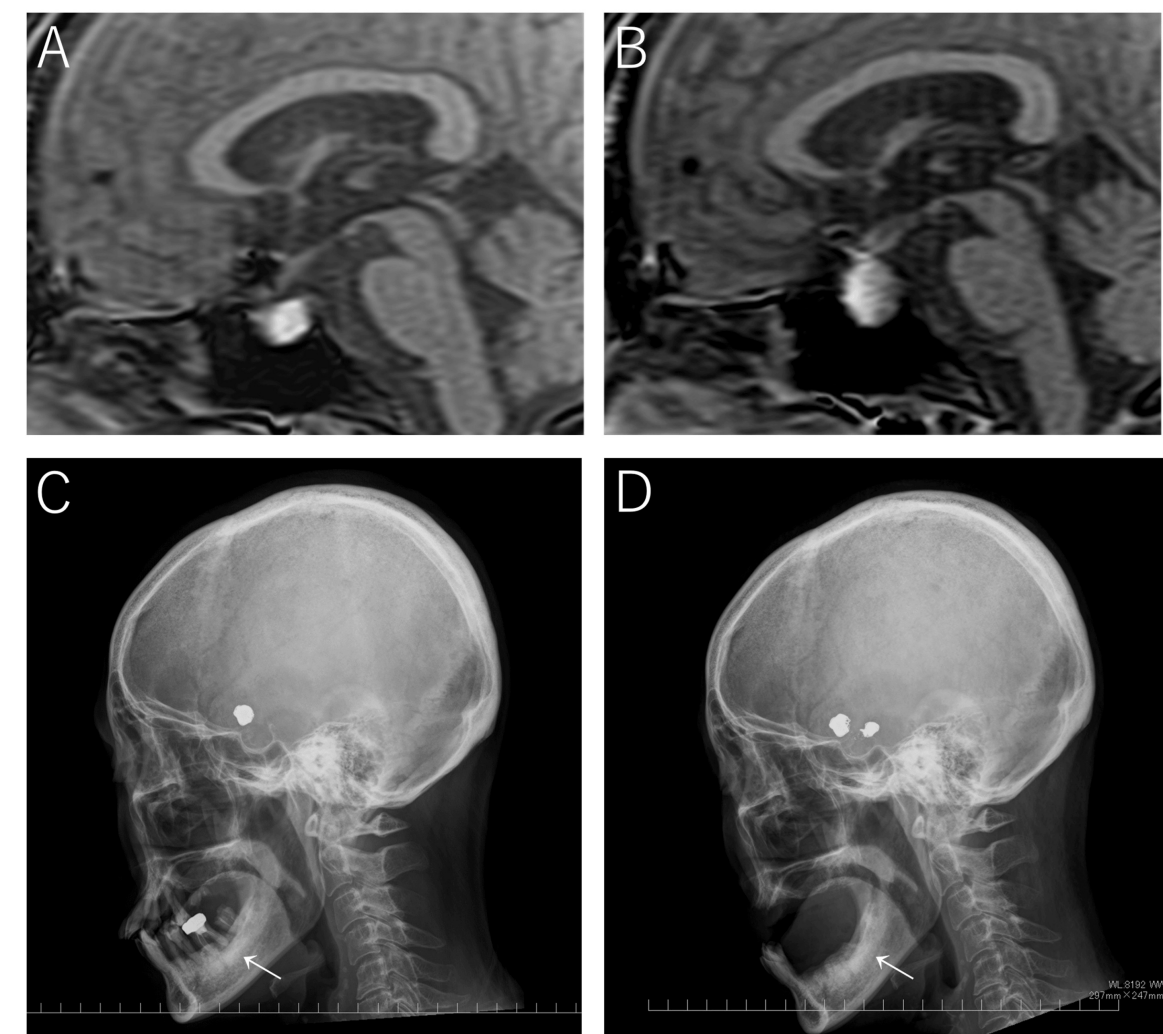
(A) Sagittal view of the magnetic resonance image showing that the pituitary gland is enlarged at postoperative month (POM) 1. (B) The size of the pituitary gland is dramatically increased at POM 7. (C) Lateral view of the cranial radiography image showing that most of the teeth were intact when the aneurysms were treated. (D) Almost all of the teeth had been extracted when the pituitary abscess was treated.

Her poor oral hygiene resulted in dental caries that were untreated for many years; therefore, tooth extraction was required. A throat culture during hospitalization revealed positive results for *Escherichia coli* (*E. coli*), which rarely occurs in the mouth. She experienced recurrent headaches after discharge. Eleven months after subarachnoid hemorrhage treatment, she developed a fever, altered consciousness, and vomiting. Further examination revealed a pituitary lesion. Retrospective brain magnetic resonance imaging at POM 1 and POM 7 showed pituitary gland enlargement. Additionally, the high signal intensity on T1-weighted imaging suggested the possible presence of a Rathke’s cleft cyst. (Figure 2). Reduction of antiplatelet medications and elective surgery were planned. During the waiting period, after several teeth were extracted, the patient experienced severe headaches, vomiting, altered consciousness, and fatigue; therefore, she was transferred to our hospital at POM 14.

Examination at the time of the second admission revealed that she was disoriented but had vital signs almost within the normal range. Neurological examinations revealed no visual disturbances or ocular movement disorders. Findings suggestive of infections such as meningitis, sinusitis, and otitis media were not detected. Most of the teeth with caries had been extracted (Figure 2). Blood test results are presented in Table 1.

**Table 1:** Laboratory tests of the patient at the second admission.

Test	Result	Reference range
Glucose	55 mg/dL	73–109
Sodium	144 mmol/L	138–145
White cell count	12,550/μL	3,300–8,600
C-reactive protein	1.3 mg/dL	0.00–0.14
Adrenocorticotropic hormone	6.8 pg/mL	10–60
Cortisol	3.12 µg/dL	7.07–19.6
Prolactin	0.96 ng/mL	7.0–40
Insulin-like growth factor Ⅰ	58 ng/mL	70–201
Thyroid-stimulating hormone	0.261 µIU/mL	0.35–4.94
Free triiodothyronine (T3)	2.38 pg/mL	1.88–3.18
Free thyroxine (T4)	0.53 pg/mL	0.70–1.48

**Figure 3:**
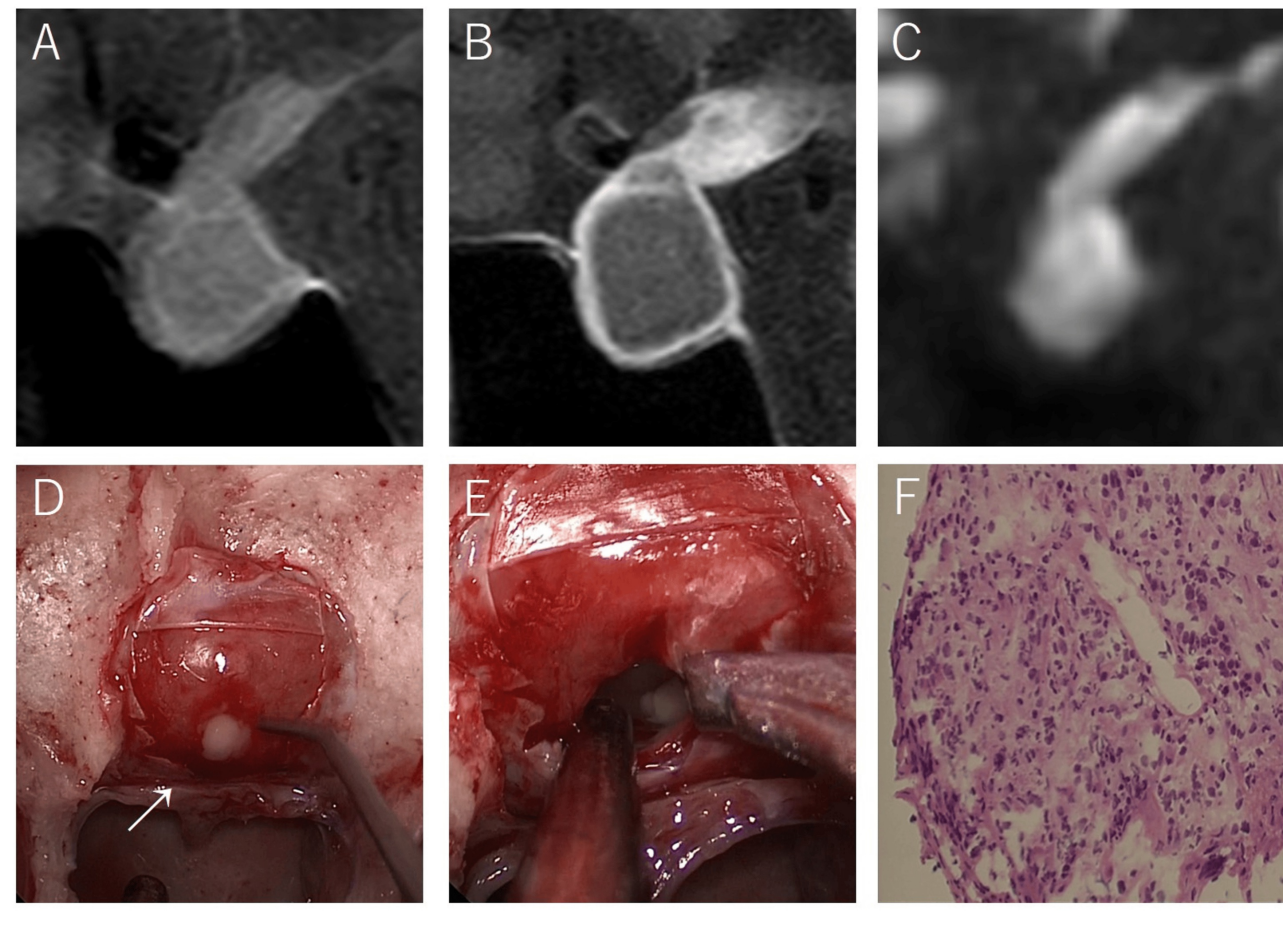
The sagittal view of brain magnetic resonance imaging showed enlargement of the pituitary gland with intermediate hyperintensity on T1-weighted imaging (A), peripheral rim of contrast enhancement on T1-weighted imaging (B), and hyperintensity on diffusion-weighted imaging (C). An abscess was observed in the pituitary gland in the intraoperative photograph (D). Large amounts of pus were drained from the sella turcica (E). Histopathological findings did not reveal any obvious tumor components but showed fibrotic foci and inflammatory exudate containing neutrophils (F).

Brain magnetic resonance imaging showed a 21 mm enlargement of the pituitary gland, which protruded partially into the right cavernous sinus that was intermediately hyperintense on both T1-weighted and diffusion-weighted imaging without a fluid-fluid level. A peripheral rim of contrast enhancement was observed on T1-weighted imaging, and T1 hyperintensity in the posterior lobe was obscured (Figure 3).

The patient was diagnosed with a pituitary abscess and hypopituitarism. Hormone replacement therapy was initiated, and surgery was planned. Before surgery, the patient became reoriented and symptoms such as headache, nausea, and fatigue improved. Blood cultures and echocardiography revealed no abnormalities, and the presence of infective endocarditis was considered unlikely. Endoscopic drainage was performed during the preoperative diagnosis of a pituitary abscess. Histopathological findings did not reveal any obvious tumor components; however, they showed fibrotic foci and inflammatory exudate containing neutrophils. The pus culture was positive for *E. coli*, and a pituitary abscess caused by *E. coli* was diagnosed. Antibiotic treatment with meropenem was initiated and switched to ceftriaxone, followed by trimethoprim/sulfamethoxazole for two weeks. Subsequently, the patient was discharged. After surgery, hormone replacement therapy for hypopituitarism was continued. Recurrence was not observed during a four-year postoperative period. The patient provided informed consent for the publication of this case.

## Discussion

Pituitary abscesses are rare and account for approximately 1% of all pituitary lesions [1,3,4]. One-third of all pituitary abscesses arise from preexisting lesions, such as Rathke’s cleft cysts, and are known as secondary pituitary abscesses [1,3-5]. The positivity rates of cultures of pituitary abscesses range from 19.7% to 61%; sometimes, the causative organisms cannot be identified because of preoperative antibiotic therapy [1,3-6]. According to Agyei et al., who summarized 200 cases of pituitary abscess, most of the causative organisms were Gram-positive cocci, which are skin-indigenous bacteria, and only one patient had positive culture results for *E. coli* [1]. Anterior pituitary dysfunction was the most common symptom (81.8%), followed by headache (69.7%), diabetes insipidus (47.9%), and visual deficits (45.5%) [3]. Although a pituitary abscess is an infectious disease, fever and an elevated white blood cell count are observed in only approximately 20% to 40% of cases [1,3-5,7]. Determining the correct preoperative diagnosis is difficult because a pituitary abscess can present with various magnetic resonance signals; additionally, pituitary abscesses are often misdiagnosed as pituitary adenoma with necrosis or cystic degeneration, pituitary apoplexy, or Rathke’s cleft cyst [4,6]. When a pituitary abscess is suspected, prompt surgical exploration and postoperative antibiotic therapy are required [1]. Approximately 70% of patients require hormone replacement therapy postoperatively [3]. Pituitary abscesses are generally caused by infections such as meningitis, sinusitis, osteomyelitis of the sella turcica, cavernous sinus thrombosis, and sepsis [1,3-5]. In this case, no such findings were observed; however, the patient had many dental caries.

Ewald et al. proposed three criteria for establishing the diagnosis of odontogenic brain abscesses: (1) no other source of infection, (2) positive causative organisms revealed by a throat culture, and (3) clinically and radiographically active periodontal disease or dental caries [8]. We considered that the infection spread from the dental caries to the pituitary gland because of the following: throat culture results were positive for *E. coli* during aneurysm treatment, as were the pus culture results of the pituitary abscess; *E. coli* is rarely the causative organism of a pituitary abscess; and the patient’s oral hygiene had deteriorated so much that almost all her teeth required extraction. Transient bacteremia frequently occurs after tooth extraction; therefore, reports of cases in which pituitary abscesses become symptomatic because of tooth extraction, as in this case, are available [5,9].

In this case, in addition to the ruptured anterior communicating aneurysm, a true posterior communicating artery aneurysm, which occurred in the posterior communicating artery itself but not at the branch point, and an anterior wall aneurysm of the internal carotid artery were observed. These aneurysms account for 0.1% to 2.8% and 1% of all intracranial aneurysms, respectively [10,11]. Both are rare aneurysms that are prone to rupture. Although it is not common for either of these aneurysms to be involved in an infection, it was inferred that some type of infection was involved in this case because they were located on the same side and close to the pituitary gland that formed the abscess.

Infectious intracranial aneurysms are rare and account for 0.5% to 6.5% of all intracranial aneurysms; additionally, they tend to be more fusiform than saccular in shape [2]. They are more common in the distal region in cases of infectious endocarditis, which is the most common cause of infectious intracranial aneurysms; however, they may occur in the proximal region in cases of non-infectious endocarditis [12]. Clear criteria have not yet been established for the diagnosis of infectious intracranial aneurysms according to the American Heart Association’s Scientific Statement; they are usually diagnosed using a combination of susceptible hosts, clinical signs, and physical findings and confirmed using radiological imaging and intraoperative findings [13]. Infectious intracranial aneurysms of the anterior communicating artery are rare; including this case, only seven cases have been reported [14-18] (Table 2).

**Table 2:** Summary of infectious aneurysms of the anterior communicating artery. *E. coli*: *Escherichia coli*; IE: infectious endocarditis; SAH: subarachnoid hemorrhage; TIA: transient ischemic attack; MCA: middle cerebral artery; ICA: internal carotid artery; PComA: posterior communicating artery

Case	Patient’s age	Patient’s sex	Pathogens	Number of aneurysms	Location of other aneurysms	Clinical presentation	Treatment	Clinical outcome	Immunocompromised status
Chun et al. (2001) [14]	9	M	Candida	2	MCA (M1)	SAH	Antibiotics	Death	Possibly immunocompromised
Chun et al. (2001) [14]	30	F	Coccidioides	1		SAH	Clipping/antibiotics	Good	Possibly immunocompromised
Brust et al. (1990) [15]	45	M	NA	6	5 MCA (distal)	IE/cellulitis of the foot/ischemic stroke	Antibiotics	Death	
Boissonneau et al. (2018) [16]	83	M	Campylobacter	1		Fever/visual deficit	Clipping/antibiotics	Good	
Allen et al. (2013) [17]	NA	NA	Coccidioides	2	MCA (M1)	Meningitis	Antibiotics	Death	HIV
Kyle et al. (2023) [18]	34	F	Pneumocystis	2	ICA-MCA	TIA	NA	Good	HIV
Our case	60	F	E. coli	3	ICA (C2), true PComA	SAH/pituitary abscess	Coiling	Good	

Regarding these cases, the mean age at occurrence was 43.5 years (range, 9-83 years), the male-to-female ratio was 1:1, the fungal ratio was 57% (4/7 cases), and the most common clinical presentation was subarachnoid hemorrhage (43%; 3/7 cases), followed by ischemic stroke (29%; 2/7 cases). All three patients who underwent surgical intervention experienced a good clinical course, but three patients who did not undergo surgical intervention died (75%; 3/4 cases). Boissonneau et al. reported that the mechanism of growth of anterior communicating artery aneurysms is secondary infection by *Campylobacter*, a Gram-negative bacterium similar to *E. coli* [16]. Regarding the infection route, hematogenous spread of inflammation from dental caries to the pituitary gland rarely occurs because of the presence of the blood-brain barrier [1]. Therefore, for this case, it was presumed that the infection spread from dental caries to the anterior communicating artery and internal carotid artery, leading to aneurysm formation. Furthermore, it is possible that the rupture of the infectious anterior communicating artery aneurysm and the resulting subarachnoid hemorrhage disrupted the blood-brain barrier, allowing the infection to reach the pituitary gland or a possible Rathke’s cleft cyst. Additionally, as reported by Shi et al., it is possible that the infection spread from the pituitary abscess to the internal carotid artery via the cavernous sinus, resulting in the development of an infectious intracranial aneurysm [19].

## Conclusions

We encountered a rare case of a pituitary abscess caused by *E. coli*, an uncommon constituent of the oral flora, in a patient with a history of multiple untreated dental caries and intracranial aneurysms. This report underscores the importance of considering dental infections as a potential source of central nervous system pathology. A thorough evaluation of dental health, early recognition of symptoms, and timely imaging are essential for accurate diagnosis and appropriate management. Clinicians should maintain a high index of suspicion for pituitary abscess and the possibility of infectious aneurysms when encountering multiple aneurysms with atypical morphology or location, particularly in patients with poor oral hygiene or a suspected Rathke’s cleft cyst. Although the exact route of infection remains unclear, it is possible that an infectious intracranial aneurysm developed in the anterior communicating artery adjacent to the pituitary gland.
